# Physicochemical Parameters Limiting Growth of *Debaryomyces hansenii* in Solutions of Hygroscopic Compounds and Their Effects on the Habitability of Martian Brines

**DOI:** 10.3390/life11111194

**Published:** 2021-11-05

**Authors:** Jacob Heinz, Vita Rambags, Dirk Schulze-Makuch

**Affiliations:** 1 Center for Astronomy and Astrophysics (ZAA), Astrobiology Research Group, Technische Universität Berlin, Hardenbergstr. 36, 10623 Berlin, Germany; v.rambags@student.maastrichtuniversity.nl (V.R.); schulze-makuch@tu-berlin.de (D.S.-M.); 2 Faculty of Science and Engineering, Maastricht University, 6211 LK Maastricht, The Netherlands; 3 German Research Centre for Geosciences (GFZ), Section Geomicrobiology, 14473 Potsdam, Germany; 4 Department of Experimental Limnology, Leibniz-Institute of Freshwater Ecology and Inland Fisheries (IGB), 16775 Stechlin, Germany; 5 School of the Environment, Washington State University, Pullman, WA 99164, USA

**Keywords:** Mars, brines, salts, microorganisms, halotolerance, yeast, microbial growth, water activity, chaotropicity

## Abstract

The availability of liquid water is a prerequisite for all lifeforms on Earth. In hyperarid subzero environments like the Dry Valleys in Antarctica or the near-subsurface of Mars liquid water might be provided temporarily by hygroscopic substances that absorb water from the atmosphere and lower the freezing point of water. To evaluate the potential of hygroscopic compounds to serve as a habitat, it is necessary to explore the microbial tolerances towards these substances and their life-limiting properties. Here we present a study investigating the tolerances of the halotolerant yeast *Debaryomyces hansenii* to various solutes. Growth experiments were conducted via counting colony forming units (CFUs) after inoculation of a liquid growth medium containing a specific solute concentration. The lowest water activities (a_w_) enabling growth were determined to be ~0.83 in glycerol and fructose-rich media. For all other solutes the growth-enabling a_w_ was higher, due to additional stress factors such as chaotropicity and ionic strength. Additionally, we found that the solute tolerances of *D. hansenii* correlate with both the eutectic freezing point depressions and the deliquescence relative humidities of the respective solutes. Our findings strongly impact our understanding of the habitability of solute-rich low a_w_ environments on Earth and beyond.

## 1. Introduction

Earth harbors a tremendous variety of microbial biotopes ranging from carbon-rich tropical forest soils with a very high biomass [1] to extreme habitats like hyperarid polar deserts comprising highly specialized microbial communities [2]. Nevertheless, our solar system provides some environments that could potentially serve as habitats, which have no analogs on Earth. Laboratory work is needed to investigate the potential habitability of such extraterrestrial environments.

A promising putative habitat might be environments which include hygroscopic compounds that can easily attract water from the atmosphere and form concentrated liquid solutions via a process called deliquescence [3]. The advantage of these water absorbing substances is that they can provide liquid water essential for life even in places of hyperaridity, as has been described for halophilic cyanobacteria thriving in salt crusts in the Atacama Desert, Chile [4]. Furthermore, the liquid solutions formed via deliquescence have a lower freezing point than pure water and can extend the range for the availability of liquid water to subzero temperatures [5]. The lowest temperature at which a solution can stay liquid is called eutectic temperature. It can be reached at a certain solute concentration known as the eutectic concentration. Hygroscopic compounds that absorb water from the atmosphere are salts such as sodium chloride (NaCl) and the even more deliquescent perchlorate (ClO_4_^−^) salts, which have been detected on Mars in relatively large quantities (0.4–0.6 wt% at the Phoenix landing site [6]), or organic substances like sugars and alcohols, both detected in comets [7]. Even gases can act as hygroscopic agents like ammonia (NH_3_), which has been hypothesized to occur in a subzero H_2_O-NH_3_ ocean beneath the surface of Saturn’s moon, Titan [8].

Life at high solute concentrations can be limited by several factors. Water activity (a_w_) is a measure for the energy status of water in a system and provides a quantitative measure of the fraction of water molecules available for biological or chemical processes. While pure water has an a_w_ of 1.0, a saturated NaCl solution at 25 °C exhibits an a_w_ of 0.755 [9]. Solutes with a higher solubility than NaCl can decrease a_w_ even further. It has been argued that there is a common a_w_ limit for life at approximately 0.61 [10] with some evidence for fungal germination at slightly lower a_w_ values of approximately 0.57 [11]. However, most solutes cannot be tolerated at a_w_ as low as ~0.6 because other physicochemical parameters limit microbial survival already at a higher a_w_. For example, there is evidence that in solutions with high ionic charges, the ionic strength can be the life-limiting factor rather than a_w_ [12]. Furthermore, chaotropicity, a value describing the entropic disordering of biomacromolecules like proteins or lipid bilayers, has been described as a major biological stressor in many environments [13,14,15]. In addition, the size and strength of hydration shells around the solutes may influence membrane permeability and, consequently, affects the toxicity to organisms [16,17]. Other solute-specific parameters may influence the toxicity as well [17], such as interaction with metabolic processes, changes of pH or an enhanced redox potential of the solutes.

In this study, we investigated the microbial habitability of solutions containing various salts or organic solutes, and here we discuss how different physicochemical parameters influence the microbial survivability. As a model study organism, we chose the halotolerant yeast *Debaryomyces hansenii*, as it provides a large metabolic toolset to enable growth at low a_w_ [18]. Furthermore, a recent study has found that eukaryotes, specifically fungi, can tolerate higher ClO_4_^−^ concentrations than bacteria and archaea, and that *D. hansenii* reveals the highest microbial ClO_4_^−^ tolerance described to date [19]. The findings of our study provide several important implications for a better understanding of life in extreme terrestrial and extraterrestrial habitats.

## 2. Materials and Methods

### 2.1. Organisms and Culture Conditions

The halotolerant yeast *D. hansenii* (DSM 3428) was obtained from the Leibniz Institute DSMZ—German Collection of Microorganisms and Cell Cultures. The yeast cells were grown aerobically and without shaking at their optimum growth temperature of 25 °C in liquid DMSZ growth medium #90 (3% malt extract, 0.3% soya peptone) containing an additional solute of interest at a specific concentration (see Section 2.2). The growth media were prepared by mixing the media components, the solute and water, followed by pH adjustment (pH ~ 5.6) and sterile filtration.

### 2.2. Determination of Solute Tolerances

A stock culture of *D. hansenii* was used to inoculate 5 mL of liquid growth medium containing a specified concentration of one of the solutes listed in Table 1. Growth or death of cells in the respective medium was determined by plating 100 µL-aliquots of the samples on DMSZ growth medium #90 agar plates (3% malt extract, 0.3% soya peptone, 1.5% agar) and counting colony forming units (CFUs) after 3–4 days of colony growth. All growth experiments were conducted as biological duplicates, i.e., for each solute concentration two separate samples were inoculated. When growth was detected via an increase of CFUs per mL of sample, the grown cell culture was used to inoculate a new sample with a higher concentration of the respective solute as described previously [20]. The minimum increase in solute concentration for each culture transfer was set to be 0.5 mol/kg. The highest solute concentration that enabled growth was defined as the solute tolerance of the organism. The lowest concentration disabling growth was termed minimum inhibitory concentration. Solute concentrations are most often provided in molality (m) values (amount of solute per weight of water, mol/kg). Where necessary, values are also given in weight percentage (mass of solute per weight of total solution, wt%), or molarity (M) values (amount of solute per volume of total solution, mol/L), which is calculated from molality values by considering the densities of the growth media provided in Table A1. Minor uncertainties in the final solute concentration in the medium might have arisen from the sample treatment (sterile filtration, pH adjustment) and from the hygroscopicity of some solutes possibly leading to the introduction of marginal amounts of additional water being absorbed by the solute. These minor potential errors were considered as negligible compared to the incremental stepwise solute concentration increases of 0.5 mol/kg.

### 2.3. Water Activity Measurements

The water activity (a_w_) was measured at 25 °C for the growth media with the highest solute concentration that enabled growth (Table 1), as well as for the minimum inhibitory concentration (Table A1). The a_w_ measurements were conducted with the Rotronics ‘HC2-AW-USB’ a_w_ meter that was calibrated before the measurements with five saturated salt solutions (MgCl_2_ ∙ 6 H_2_O, a_w_ = 0.325; Mg(NO_3_)_2_ ∙ 6 H_2_O, a_w_ = 0.530; NaCl, a_w_ = 0.755; KCl, a_w_ = 0.850; K_2_SO_4_, a_w_ = 0.975) equilibrated at 25 °C for three weeks as described by Winston and Bates, 1960 [9]. After determining the a_w_ of all growth media, the five saturated standard salt solutions were measured again to ensure reproducibility. The instrumental error of the a_w_ meter was determined to be ≤0.003.

## 3. Results

**Figure 1:**
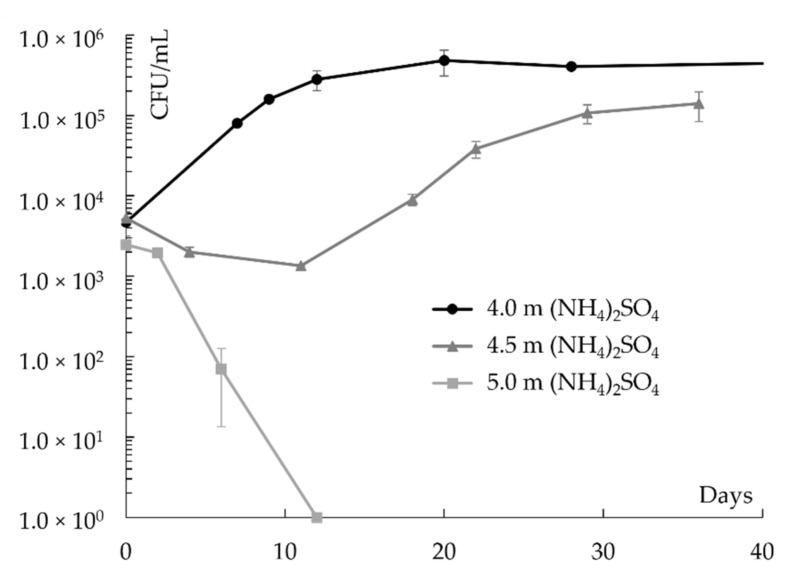
Exemplary growth curves of *D. hansenii* in NH_4_(SO_4_)_2_-containing growth media where 4.5 m and 5.0 m NH_4_(SO_4_)_2_ represent the highest NH_4_(SO_4_)_2_ concentration enabling growth and the minimum inhibitory NH_4_(SO_4_)_2_ concentration, respectively (*n* = 2).

The solute tolerances of *D. hansenii* were determined from growth experiments. Three exemplary growth curves in NH_4_(SO_4_)_2_-containing growth media are shown in Figure 1.

In the first sample (black line and circles), containing 4.0 m NH_4_(SO_4_)_2_, robust growth was detected without a notable lag phase, while in the second sample (dark gray line and triangles), containing 4.5 m NH_4_(SO_4_)_2_ and having an a_w_ of 0.864 (Table 1), a long lag phase of more than 10 days and a reduced growth rate indicated that the salt concentration was close to the actual maximum NH_4_(SO_4_)_2_ concentration tolerated by *D. hansenii*. Consequently, in the third sample (light grey line and squares), containing 5.0 m NH_4_(SO_4_)_2_ and having an a_w_ of 0.842 (Table A1), only cell death was observed. The second and the third sample are defined as the maximum NH_4_(SO_4_)_2_ concentration enabling growth and the minimum inhibitory concentration, respectively.

**Figure A1:**
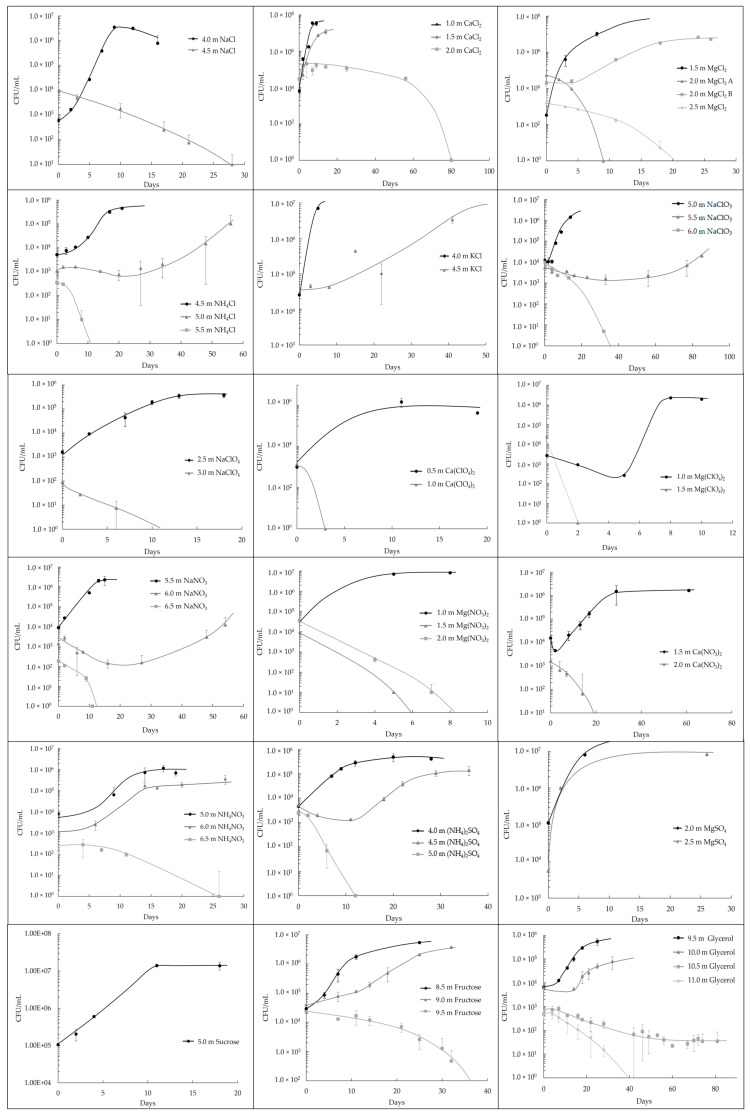
Growth curves of *D. hansenii* obtained as described in the main text in liquid growth media containing different concentrations of the solutes listed in Table 1 (*n* = 2).

The determination of the remaining solute tolerances of *D. hansenii* was conducted in the same manner. Respective growth curves were obtained also for all other solutes investigated in this study (Figure A1). The solute tolerances obtained from these growth experiments are displayed in Table 1 and are compared to values from other references. Furthermore, the a_w_ values and ionic strengths at the solute tolerance concentrations are provided.

*D. hansenii* showed the highest microbial solute tolerances reported to date to NH_4_Cl (5.0 mol/kg), NaClO_3_ (5.5 mol/kg), NaClO_4_ (2.5 mol/kg), Ca(ClO_4_)_2_ (0.5 mol/kg), Mg(ClO_4_)_2_ (1.0 mol/kg), NaNO_3_ (6.0 mol/kg), Ca(NO_3_)_2_ (1.5 mol/kg), Mg(NO_3_)_2_ (1.0 mol/kg), and (NH_4_)_2_SO_4_ (4.5 mol/kg). It can grow in saturated solutions of KCl, MgSO_4_, and sucrose at 25 °C.

The lowest a_w_ at which *D. hansenii* showed growth was ~0.83 and was observed in growth media containing 10.0 m Glycerol (a_w_ = 0.827) or 9.0 m fructose (a_w_ = 0.828). It should be noted at this point that growth at this low a_w_ (~0.83) could theoretically also occur in samples containing MgCl_2_ and NaClO_3_. The reason for this is that the minimum inhibitory concentrations for those two salts are below an a_w_ of ~0.83 (Table A1), i.e., growth might be detected at lower a_w_ when the solute concentration is increased at smaller incremental steps than 0.5 mol/kg. However, we estimate this possibility as not likely because the growth curves for media containing 5.5 m NaClO_3_ (highest concentration enabling growth) indicated only very slow growth with a lag phase of more than 50 days and growth curves for media containing 2.0 m MgCl_2_ (highest concentration enabling growth) indicated slow growth only for one of the biological duplicates while cells in the other sample died (Figure A1). This shows that both sample types are very close to their actual maximum solute concentration enabling growth, and growth of *D. hansenii* at lower a_w_ values seems to be very unlikely.

## 4. Discussion

### 4.1. Growth-Limiting Parameters

**Figure 2:**
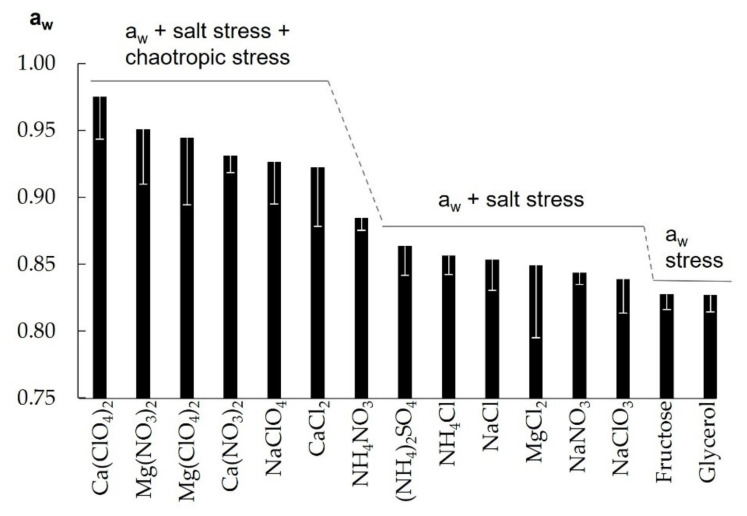
Water activities (a_w_) of growth media with highest solute concentration enabling growth (not including sucrose, KCl, and MgSO_4_, which are tolerated by *D. hansenii* at their saturation limits). White error bars indicate difference between a_w_ at highest solute concentration enabling growth (Table 1) and at the minimum inhibitory concentration (Table A1). Stressors (a_w_, salt stress, chaotropicity) interpreted to contribute to the growth limitation of *D. hansenii* are noted above the respective solutes.

This is the first study providing solute tolerances and the concomitant a_w_ values for *D. hansenii* for such a high number of different solutes (Table 1). The a_w_ values for samples not reaching solute saturation (i.e., all samples except those containing KCl, MgSO_4_, and sucrose) are plotted as bar charts in Figure 2. The lowest a_w_ at which *D. hansenii* showed growth was achieved in growth medium containing 10.0 m glycerol (a_w_ = 0.827) and 9.0 m fructose (a_w_ = 0.828). As these two solutes are both non-ionic and non-toxic organic compounds with only minor chaotropic activity [33], it is likely that in this case the a_w_ is the main limitation to the growth of *D. hansenii*. An earlier study found an a_w_ limit of 0.803 for growth of *D. hansenii* at 37 °C in medium containing yeast extract, casamino acids, and trisodium citrate broth supplemented with 3.5 M NaCl and 0.4 M MgCl_2_ [10]. The difference in the limiting a_w_ values very likely arises from the fact that two different *D. hansenii* strains were investigated (strain DSM 3428 in this study, and strain DSM 70590 in reference [10]) or (possibly additionally) from the different incubation temperatures (25 °C in this study, and 37 °C in reference [10]) and growth media compositions.

All investigated solutes other than glycerol, fructose, and sucrose (which is tolerated by *D. hansenii* at its saturation concentration) are salts which impose additional salt stress through ionic strength which has been shown to have a significant effect on the survivability of microorganisms [12]. Hence, a_w_ enabling growth of *D. hansenii* is slightly higher in salt-containing growth media compared to glycerol- or fructose-enriched media. Ionic strength might even be the major limiting factor in regard to (NH_4_)_2_SO_4_ containing samples, because they exhibit the highest ionic strength values of all investigated samples. The ionic strength in the growth medium with the highest (NH_4_)_2_SO_4_ concentration (4.0 mol/kg) enabling growth was 13.5 mol/kg (~10.2 mol/L). This is strikingly close to values in a previous study that found a growth-limiting ionic strength for most of the investigated brines at approx. 10 mol/L with only one exception, where growth occurred also at 12.1 mol/L [12].

When salts, additionally to their intrinsic ionic strength, exhibit a certain chaotropic activity, the a_w_ required for growth is significantly increased (Figure 2). Quantitative chaotropicity data is lacking for most of the salts investigated in this study, especially for perchlorates, chlorates, and most of the nitrates [33]. However, chaotropicity shows some correlation to the Hofmeister series [33,34] which orders ions in order of their ability to stabilize (kosmotropic, “salting-out” substances) or destabilize (chaotropic, “salting-in” substances) biomacromolecules such as proteins [35]. In this context, chaotropic anions are large and polarizable, and interact with the biomolecule surface via nonlocalized attractive dispersion forces, while chaotropic cations are hard and polyvalent, and bind to Lewis basic sites to the macromolecules [36]. According to this series the anions used in this study can be ordered with increasing chaotropicity (with SO_4_^2−^ being considered as strongly kosmotropic) as follows:

SO_4_^2−^ < Cl^−^ < NO_3_^−^ < ClO_3_^−^ < ClO_4_^−^ (1)

while for the cations this is:

NH_4_^+^ < K^+^ < Na^+^ < Mg^2+^ < Ca^2+^ (2)

Indeed, the salt containing the ions with the highest chaotropicity (Ca(ClO_4_)_2_) shows the lowest microbial solute tolerance and requires the highest a_w_ for enabling growth of *D. hansenii*. In a similar pattern, all perchlorates and most of the Mg^2+^ and Ca^2+^ salts enable growth of *D. hansenii* only at an increased a_w_ (Figure 2). An exception forms MgCl_2_, which has been described previously to limit survival predominately through its chaotropic activity [14]. In contrast, we found here that MgCl_2_ could be tolerated at an a_w_ comparable to the non-chaotropic NaCl. Mg^2+^-containing salts exhibited significant chaotropic stress only in combination with the more chaotropic anions NO_3_^−^ and ClO_4_^−^.

The individual contributions of the different stressors (a_w_, salt stress, chaotropicity) to the overall toxicity of a specific solute are shown in Figure 2 for better visualization in a steplike arrangement, however, the transition between the different stress regimes is most likely fluent and the precise contribution of each stressor cannot be determined exactly but can only be tendentially estimated.

While in our study ionic strength-induced salt stress, chaotropicity, and a_w_ represent the major growth-limiting factors, the ionic species of the investigated salts seem to play no or only a minor role for the solute toxicity because every type of ion is tolerated by *D. hansenii* at a large range of concentrations depending on the type of salt (Table 1). For example, chloride can be tolerated up to 3 mol/kg in CaCl_2_, 4 mol/kg in NaCl and MgCl_2_, 4.5 mol/kg in KCl (saturation limit), and 5 mol/kg in NH_4_Cl.

Another parameter that theoretically could influence the toxicity of solutes is their oxidative character. Perchlorate has a standard reduction potential of 1.39 V in acidic solutions when reduced to Cl^−^ [37] and is therefore often described as a strong oxidant. However, this is only true in the solid state and upon heating. In solution and under ambient temperatures the reduction of perchlorate is kinetically hindered and, hence, dissolved ClO_4_^−^ is astonishingly inert [38]. Furthermore, the standard reduction potential of chlorate is even higher (1.45 V in acidic solution [37]), even though the tolerance of *D. hansenii* to NaClO_3_ was more than two times higher than to NaClO_4_. That indicates that chaotropicity is a more critical parameter for cellular tolerance than the oxidative character of perchlorate.

Additional to a_w_, salt stress, and chaotropicity, some of the ions might interfere with the cell metabolism and, hence, cause additional ion-specific stresses. For example, calcium ions can act as an enzyme cofactor and as a secondary messenger in various signal transduction pathways, and are involved in the folding and processing of secretory proteins [39]. Thus, an intracellular excess of calcium ions might negatively influence these cellular pathways end increase the toxicity of calcium-containing salts. The investigation of such ion-specific interferences requires further examination and is outside of the scope of this study.

### 4.2. Implications for the Habitability of Martian Brines

Various types of salts have been detected on Mars and in Martian meteorites. Among those are sulfates [40], nitrates [41,42], bromides [43], chlorides [6], chlorates [42], and perchlorates [6]. The latter two are of special interest for this study as natural occurrences of these two salt types are relatively rare on Earth and are restricted to hyperarid environments [44,45], while perchlorates are known to occur in higher concentrations and widely distributed only on Mars [6,46]. Chlorates are likely associated with perchlorates [47] and may have even greater potential to form liquid brines on Mars than perchlorates [48]. In this context, it is notable that we found *D. hansenii* to exhibit the highest microbial tolerance reported to date to NaClO_4_ (2.5 mol/kg, slightly higher than previously reported [19]) and to NaClO_3_ (5.5 mol/kg, significantly higher than previously reported [25]). The high tolerance to NaClO_3_ especially, with an a_w_ of 0.839 being relatively close to the lower a_w_ limit of *D. hansenii* found in this study (0.827), indicates that organisms even more halophilic than *D. hansenii* might be able to tolerate chlorates at concentrations closer to the absolute a_w_ limit for life (~0.57 [11]) and, hence, to thrive in very concentrated chlorate brines. The tolerances to magnesium and calcium chlorates, though, have to be investigated in future experiments because these two salts were not available at the time of this study.

**Figure 3:**
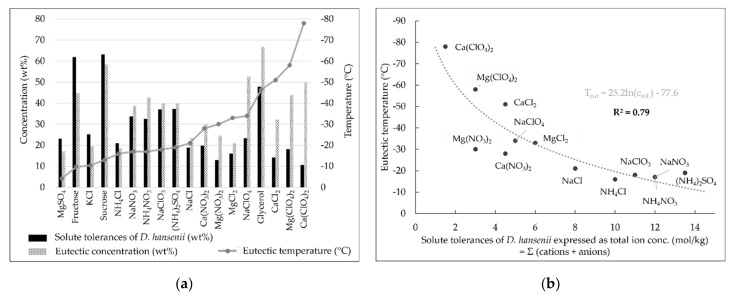
Correlation between solute tolerances of *D. hansenii* and the eutectic points of those solutes. (**a**) Comparison between solute tolerances of *D. hansenii* and eutectic concentrations as well as eutectic temperatures. It appears that the lower the eutectic freezing point of a solute the greater the discrepancy between eutectic concentration and solute tolerance of *D. hansenii*. (**b**) Logarithmic correlation between solute tolerances (total ion concentrations) of *D. hansenii* and eutectic temperatures of the investigated inorganic salts (excluding those that enabled growth at saturation concentrations at 25 °C).

Of particular interest for the habitability of subzero environments are the eutectic points of the solute-water systems as they provide information upon the maximum freezing point depressions of the solutions and the corresponding solute concentration needed. Our results reveal that *D. hansenii* can grow at eutectic concentrations of fructose, sucrose, MgSO_4_, KCl, and NH_4_Cl (Figure 3a). All of these solutes have eutectic temperatures above or equal to −16 °C (Table A1). Furthermore, the discrepancy between solute tolerance and eutectic concentration is equal or less than 5 wt% for NaNO_3_, NaClO_3_, NaCl, (NH_4_)_2_SO_4_, and MgCl_2_, suggesting that other halophilic organisms might be able to tolerate eutectic concentrations of those solutes. These five solutes span a range of eutectic temperatures from −17 °C (NaNO_3_) to −33 °C (MgCl_2_) (Table A1). It has been argued that microbial growth is presumably possible at temperatures down to approximately −20 °C [49].

It should be noted that the solutes revealing the highest discrepancy between solute tolerance and eutectic concentration are the compounds exhibiting the lowest eutectic temperatures (Figure 3a). When only the inorganic salts with a tolerance of *D. hansenii* below the saturation concentration at 25 °C are considered, a logarithmic correlation between the total ion concentration tolerance (sum of cations and anions) and the eutectic temperature of the respective salt can be found (Figure 3b), confirming that solutes causing a high freezing point depression tend to be less tolerated by *D. hansenii*. We propose that this trend is also valid for other organisms, which should prompt further investigations. The reason for the observed correlation may lay again in the chaotropicity of some of the salts. Chaotropic substances are usually considered as water structure-breaking (‘chaos-making’) [36,50]. For that reason, they have high solubilities and lower the freezing point of water more significantly than most kosmotropic substances. As noted above, the same chaotropic activity decreases the stability of macromolecular biomolecules and limits survival of microorganisms.

**Figure 4:**
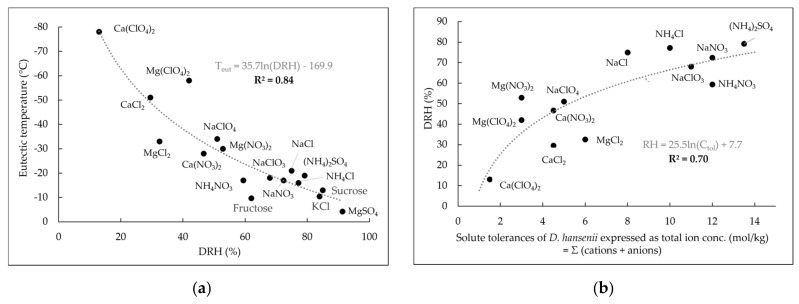
(**a**) Logarithmic correlation between eutectic temperatures of solutes and their deliquescence relative humidities (DRH). (**b**) Logarithmic correlation between the DRH of the solutes and the solute tolerances of *D. hansenii* (total ion concentrations) for all ionic species with maximum concentrations enabling growth below saturation concentration at 25 °C.

On top of that, the eutectic temperature correlates also with the solute’s deliquescence relative humidity (DRH, Table A1) which is the relative humidity at which a substance starts to absorb water from the atmosphere (Figure 4a). Hence, the DRH is a measure for the hygroscopicity of a compound. Our results show in good approximation that the more hygroscopic (i.e., lower DRH) a solute, the lower is the tolerance of *D. hansenii* towards this solute (Figure 4b). Even though more research is needed to confirm these findings, the described correlations indicate that the most hygroscopic and freezing point-depressing and, hence, most promising candidates for providing liquid water under the subzero conditions of Mars, are also the substances causing the highest stress levels to microorganisms due to high solubilities (coinciding with low a_w_) and enhanced chaotropicity.

## 5. Conclusions

In this study we determined the tolerances of *D. hansenii* for several solutes. We found evidence for three parameters limiting the survival of *D. hansenii* at high solute concentrations. For non-toxic, non-, or slightly chaotropic organic solutes such as glycerol and fructose the a_w_ in the growth medium seems to be the major growth-limiting factor. Salt species additionally exhibit salinity stress caused by their intrinsic ionic strength, which increases the minimum a_w_ necessary for growth of *D. hansenii*. The minimum a_w_ required for growth is significantly more increased when the solutes show additionally an enhanced chaotropic activity.

Even though we found noticeable high tolerances of *D. hansenii* to various salts occurring on Mars, our findings indicate that the capability of a hygroscopic substance to absorb water and to lower the freezing point of water negatively correlates with the microbial tolerance towards that solute. This lowers the potential of very hygroscopic compounds like calcium or magnesium perchlorates to serve as a water-providing habitat for halophilic microorganisms to some degree. Nevertheless, more research is needed to confirm these findings. For example, we suggest testing whether kosmotropic compounds can significantly compensate the environmental stress caused by chaotropic substances as previous studies suggested [51,52]. Furthermore, investigating the temperature dependence of the here reported microbial solute tolerances could confirm the previously reported findings that lower temperatures increase the tolerance to some solutes [20,53] and that chaotropic substances can expand microbial growth windows at subzero temperatures [54].

## Figures and Tables

**Table 1:** Solute tolerances of *D. hansenii* compared with literature data (for organisms other than *D. hansenii* only one representative example is provided), as well as measured water activities (a_w_) and calculated ionic strengths (I) at the respective solute concentration. Values in parentheses are at or very close to saturation of the respective solutes and, hence, higher tolerances (and lower a_w_ values) might be possible theoretically but were not detectable.

Solute	Solute Tolerance			I	a_w_	Literature Solute Tolerances [mol/L]	
	[mol/kg]	[mol/L]	[wt%]	[mol/kg]		*D. hansenii*	Other Organisms ^a^
**Salts**							
NaCl	4.0	3.6	18.9	4	0.854	4.0 [21]	6.1 ^c^ Hs [22]
NH_4_Cl	5.0	4.1	21.1	5	0.856	–	1.5 M [23]
CaCl_2_	1.5	1.4	14.3	4.5	0.922	–	2.1 Ec [24]
MgCl_2_	2.0	1.9	16.0	6	0.849	–	2.0 Af [24]
KCl	(4.5)	(3.9)	(25.1)	(4.5)	(0.852)	4.0 [21]	4.0 ^c^ Aw [15]
NaClO_3_	5.5	4.5	36.9	5.5	0.839	–	2.8 Hv [25]
NaClO_4_	2.5	2.2	23.4	2.5	0.926	2.1 [19] ^b^	1.1 Ph [20]
Ca(ClO_4_)_2_	0.5	0.5	10.7	1.5	0.975	–	0.1 Ph [20]
Mg(ClO_4_)_2_	1.0	0.9	18.2	3	0.944	–	0.3 Hl [26]
NaNO_3_	6.0	4.9	33.8	6	0.844	–	3.5 Mv [27]
NH_4_NO_3_	6.0	4.6	32.4	6	0.880	–	5.2 JH [28]
Ca(NO_3_)_2_	1.5	1.4	19.8	4.5	0.931	–	–
Mg(NO_3_)_2_	1.0	1.0	12.9	3	0.944	–	–
(NH_4_)_2_SO_4_	4.5	3.4	37.3	13.5	0.864	–	1.0 Cg [29]
MgSO_4_	(2.5)	(2.4)	(23.1)	(10)	(0.946)	–	2.7 ^c^ H [30]
**Organic solutes**							
Sucrose	(5)	(2.4)	(63.1)	–	(0.872)	~1.3 [31]	2.3 Ea [28]
Fructose	9.0	4.4	61.9	–	0.828	–	4.4 Ea [28]
Glycerol	10.0	5.4	47.9	–	0.827	≥2.5 [32]	7.6 Xb [28]

^a^ Organisms abbreviations: Hs—*Halobacterium salinarum*; M—*Methanosarcina* spp.; Ec—*Eurotium chevalieri*; Af—*Aspergillus flavipes*; Hv—*Halomonas venusta*; Ph—*Planococcus halocryophilus*; Hl—*Halorubrum lacusprofundi*; Mv—*Micrococcus* varians subsp. *halophilus*; Cg—*Corynebacterium glutamicum*; JH—strain JH06THJ isolated from an antique wooden artefact (Thailand); H—*Halomonas* spp.; Xb—*Xeromyces bisporus*; Ea—*Eurotium amstelodami*; Aw—*Aspergillus wentii*. ^b^ value calculated from 23 wt% NaClO_4_ [19], corresponding to 2.4 mol/kg. ^c^ saturation concentration at room temperature.

## Data Availability

All data is contained within the article.

## Appendix B

**Table A1:** Solute tolerances (C_tol_) of *D. hansenii* (see Table 1) and corresponding growth media densities achieved from weight per volume ratio of the media. Furthermore, minimum inhibitory concentrations (MIC) and the corresponding growth medium water activities (a_w_) are provided. Values in brackets are at or close to saturation concentration of the respective solutes and, hence, no MIC can be determined. Additionally, values for the eutectic points and the deliquescence relative humidities (DRH) at 20 or 25 °C are provided for all solutes.

Solute	C_tol_	Density at C_tol_	MIC	a_w_ at MIC	Eutectic Point [5,55,56,57,58,59]		DRH [9,60,61,62,63,64]
	[mol/kg]	[g/mL]	[mol/kg]		Conc. [wt%]	Temp. [°C]	[%]
**Salts**							
NaCl	4.0	1.12	4.5	0.830	23.3	−21	75
NH_4_Cl	5.0	1.05	5.5	0.842	18.7	−16	77
CaCl_2_	1.5	1.08	2.0	0.878	32.3	−51	30
MgCl_2_	2.0	1.12	2.5	0.795	21.0	−33	33
KCl	(4.5)	1.15	–	–	19.7	−10	84
NaClO_3_	5.5	1.29	6.0	0.813	39.8	−18	68
NaClO_4_	2.5	1.14	3.0	0.895	52.7	−34	51
Ca(ClO_4_)_2_	0.5	1.05	1.0	0.943	50.1	−78	13
Mg(ClO_4_)_2_	1.0	1.10	1.5	0.894	43.9	−58	42
NaNO_3_	6.0	1.22	6.5	0.835	38.6	−17	72
NH_4_NO_3_	6.0	1.13	6.5	0.875	42.7	−17	59
Ca(NO_3_)_2_	1.5	1.15	2.0	0.919	29.9	−28	47
Mg(NO_3_)_2_	1.0	1.10	1.5	0.910	24.6	−30	53
(NH_4_)_2_SO_4_	4.5	1.20	5.0	0.842	39.8	−19	79
MgSO_4_	(2.5)	1.25	–	–	17.0	−4	91
**Organic solutes**							
Sucrose	(5)	1.28	–	–	58.5	−13	85
Fructose	9.0	1.28	9.5	0.816	44.7	−10	62
Glycerol	10.0	1.12	11.0 ^a^	0.814	66.7	−47	–

^a^ A sample containing 10.5 m Glycerol (a_w_ = 0.820) was also investigated, however, it showed neither growth nor complete death of cells within the experimental time frame (Figure A1).
